# Room Temperature Ammonia Gas Sensing Using Mixed Conductor based TEMPOS Structures

**DOI:** 10.3390/s8106355

**Published:** 2008-10-14

**Authors:** Mamta Saroch, Sunita Srivastava, Dietmar Fink, Amita Chandra

**Affiliations:** 1 Department of Physics, Panjab University, 160014 Chandigarh, India; E-mails: mamtasaroch@yahoo.co.in; sunita@pu.ac.in; 2 Hahn-Meitner-Institute, Glienicker Str. 100, D-14109 Berlin, Germany; E-mail: fink@hmi.de; 3 Department of Physics and Astrophysics, University of Delhi, 110007 Delhi, India;

**Keywords:** Etched ion tracks, TEMPOS structures, ammonia sensors, impedance measurements

## Abstract

The current/voltage characteristics of mixed (ion+electron) conductor-based ‘TEMPOS’ (**T**unable **E**lectronic **M**aterial with **P**ores in **O**xide on **S**ilicon) structures̵ are reported. TEMPOS are novel electronic MOS-like structures having etched swift heavy ion tracks (i.e., nanopores) in the dielectric layer filled with some conducting material. The three contacts (two on top and one on the bottom), which resemble the classical bipolar or field effect transistor arrangements are, in principle, interchangeable when the overall electrical resistance along the tracks and on the surface are similar. Consequently, three configurations are obtained by interchanging the top contacts with the base contact in electronic circuits. The current/voltage characteristics show a diode like behaviour. Impedance measurements have been made for TEMPOS structures with tracks filled with ion conductors and also mixed conductors to study the ammonia sensing behaviour. The impedance has been found to be a function of frequency and magnitude of the applied signal and concentration of the ammonia solution. This is attributed to the large number of charge carriers (here protons) available for conduction on exposure to ammonia and also to the large surface to volume ratio of the polymer composites embedded in the ion tracks. The measurement of both, the real and imaginary parts of impedance allows one to enhance the detection sensitivity greatly.

## 1. Introduction

### 1.1. Ammonia sensors

Ammonia is widely used to synthesize various materials in chemical industries. It is a colourless gas with a special odour and is very harmful to the human body. Ammonia is a highly toxic agent, as it has the potential to harm tissues and affect the immune system. It may inhibit growth in cell lines [1, 2]. It can be produced in deteriorating/decomposing food/fruitbodies by a variety of micro and macro-fungi [1-4]. Thus, the detection and measurement of ammonia in the atmosphere is an extremely important issue with implications to the environment and medical practice as well as the automotive and chemical industries [5, 6]. Our present investigation deals mainly with the detection of ammonia by an ion track – based device using TEMPOS (**T**unable **E**lectronic **M**aterial with **P**ores in **O**xide on **S**ilicon) structures.

There are many principles for measuring both, gaseous and liquid phase ammonia described in literature. The most frequently used techniques in commercial gaseous ammonia detectors are conducting polymer ammonia analyzers, metal-oxide ammonia sensors, catalytic ammonia detectors, and optical ammonia detection techniques [6]. Ammonia sensors based on polymers – polyaniline [5, 7], Nafion [8] and polypyrrole [9] thin film coatings – have been reported. The ammonia sensing mechanism in polypyrrole (see Table 1) is twofold: an irreversible reaction between ammonia and the polymer followed by the reversible reduction of the oxidized form of the polymer causing a change in conductivity. The lower detection limit is about 1 ppm and response times of about 4 min have been recorded [6]. A decrease in sensitivity over time on exposure to ammonia is the major drawback of the polymer-based sensors.

Ammonia sensors based on metal oxides SnO_2_ [10, 11], CdSnO_3_ [12], TiO_2_ [13], In_2_O_3_ [14], and many others have been studied in the past. It has been proven that these gas sensors operate on the principle of conductance change due to chemisorption of gas molecules to the sensing layer [15]. The metal-oxide films consist of a large number of grains, and therefore, grain boundaries [16]. On exposure to a chemically reducing gas, like ammonia, co-adsorption and mutual interaction between the gas and the oxygen result in oxidation of the gas at the surface. This eventually causes removal of oxygen from grain surface leading to a decrease in the double Schottky potential barrier at the interface of adjacent grains. This leads to a change in conductivity. The lowest ammonia detection limit found in literature is 1 ppm [17] and response time is ∼ 1 minute [18]. The major drawback of metal oxide-based sensors is their lack of selectivity to one particular gas. Addition of metals or additives enhances the chemisorption of specific gases. Another straightforward ammonia sensing technique in humid ambient operates via detection of pH variation [5] via the Henderson-Hassellbach equation. However, the response depends on the source of ammonia (e.g., the types of ammonia producing fungus).

Catalytic ammonia sensors have response time ∼1s and lower detection limit as 1 ppm whereas the optical ammonia sensors have response time as 1 s and limit as 1ppb [6]. Insulating as well as conducting polymers can be used for gaseous/liquid ammonia detection. Bisphenol A polycarbonate with etched ion tracks [19] is a characteristic example of the first group and swift heavy ion modified polymer-conducting polymer composites (IPCP) also act as ammonia sensors with response time of 8 minutes for 1000 ppm [20].

### 1.2. TEMPOS structures

**Figure 1.**
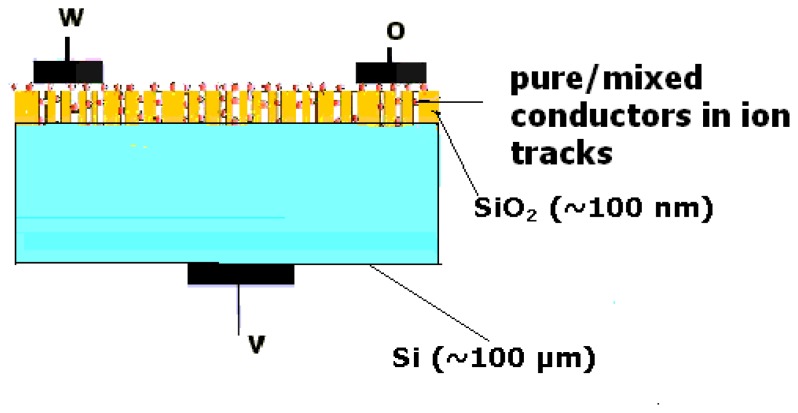
Schematic representation of a TEMPOS structure.

Our interest lies in the room temperature sensing of gaseous ammonia using an ion track-based device called ‘TEMPOS’-Tunable Electronic Materials with Pores in Oxide on Silicon [21 – 27] having etched ion tracks inserted with ionic and mixed conductors [28 – 31]. TEMPOS structures (Figure 1) consist of a dielectric layer (silicon dioxide) on a semiconductor substrate (silicon). The dielectric layer is irradiated with swift heavy ions (here, 350 MeV Au^26+^) so that individual latent tracks are formed. These tracks are then etched with a suitable etchant (here, 0.55% HF) to create parallel open pores into which mixed conductors are inserted [29–32]. Applications of TEMPOS structures, including sensors, are given in [32]. The gas sensing behaviour of a given sensing element depends on the material used, the method of preparation, and the resulting microstructure [33–37]. The mixed conductors used here were CdS or PbS, or ZnS dispersed polymer electrolyte (PEO:NH_4_ClO_4_). This specific polymer electrolyte has been chosen because it is a known proton conductor and reacts to H^+^ ion containing impurities (humidity, ammonia etc.). Finally, on this structure metallic contacts are deposited on the front (denoted here as ***o*** and ***w***) and rear (***v***) sides, according to Figure 1.

TEMPOS structures have already been found to be good humidity sensors [38]. The current/voltage characteristics of ionic and mixed (ion+electron) conductor-based TEMPOS are reported in this paper. In the present study, a PEO:NH_4_ClO_4_ +CdS (96:4+5 wt.% of Cd-salt) sample was used. The NH_4_ClO_4_ salt complexes with the ether oxygen of the polymer chain while the NH_4_^+^ ion hangs out. When the polymer chain breathes in-out, the H^+^ ions can jump to energetically equivalent sites, thereby giving rise to H^+^ ion conduction. The concentration chosen here yields the optimum conduction of the polymer electrolyte. As this is deposited as a thin layer on the ion track walls, it has a large surface area. Therefore, exposure to NH_3_ increases the number of H^+^ ions, thereby enhancing the conductivity. Also, if a lower or higher ratio of PEO: NH_4_ClO_4_ were taken, the conductivity would be so low that a sufficient number of energetically equivalent sites would not be available for H^+^ ions to jump and conduct.

### 1.3. Ion/Mixed conductor

Polymer electrolytes are ion conductors formed by complexing dissociable salts like ammonium iodide, ammonium perchlorate, etc. with a polar polymer [polyethylene oxide (PEO), polypropylene oxide (PPO), etc.]. Mixed (ion + electron) conducting polymers are obtained by the dispersal of semiconductor like CdS, PbS, ZnS, etc. in polymer electrolyte such as PEO:NH_4_ClO_4_. This introduces substantial electronic conductivity in an otherwise pure ionic conductor, which is thus an ideal electrode material for solid-state devices [29 – 32].

### 1.4. Techniques used for ammonia sensing

Detection of gases is generally carried out by measurement of the dc resistance of films. The sample resistance measured using dc current has contributions from different regions of the sample such as intragrain areas, grain boundaries and electrode-sample interface. As these contributions cannot, in the general case, be separated from each other, impedance spectroscopy is additionally employed here [33-37]. The ability to gather various kinetic and mechanistic information has made impedance spectroscopy a favorite tool for materials characterization. It involves the measurement of the impedance Z with respect to the frequency which can give details about the physical processes going on in the material through their electrical analogue. Its advantage lies in isolating individual reaction/migration steps of a multistep process. That is, since each reaction or migration step has, ideally a unique time constant associated with it, these steps can be separated in the frequency domain. Impedance spectroscopy has been employed here to study the ammonia sensing behaviour of TEMPOS structures with pure/mixed conductors.

## 2. Results and Discussion

**Figure 2.**
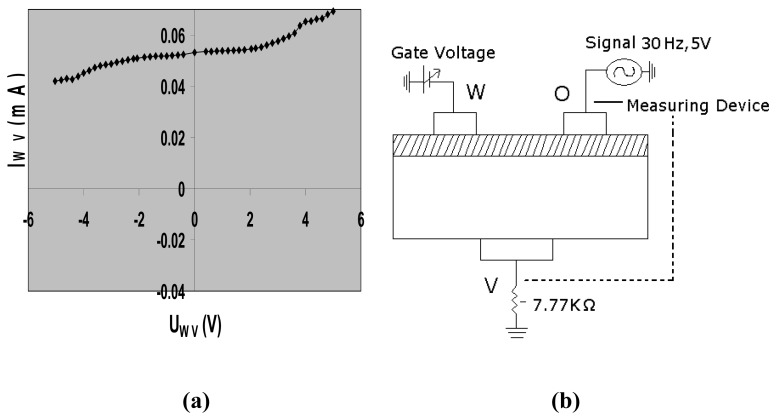
(a) Current/voltage characteristics of a CdS based TEMPOS structure in configuration 1; (b) the principle measuring setup for the current/voltage determination in configuration 1.

**Figure 3.**
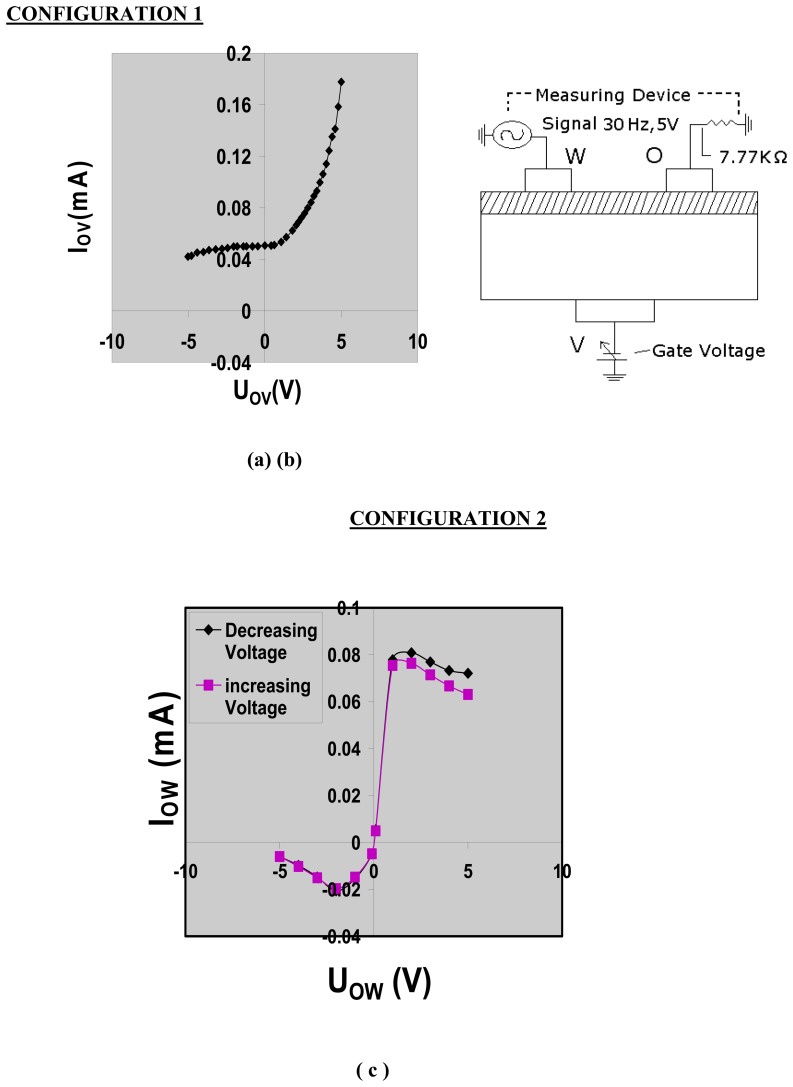
(a) Current/voltage characteristics of CdS based TEMPOS structures in configuration 2; (b) the principle measuring setup for the current/ voltage determination in configuration 2; (c) current/voltage characteristics of CdS based TEMPOS structures in configuration 2, with floating gate.

**Figure 4.**
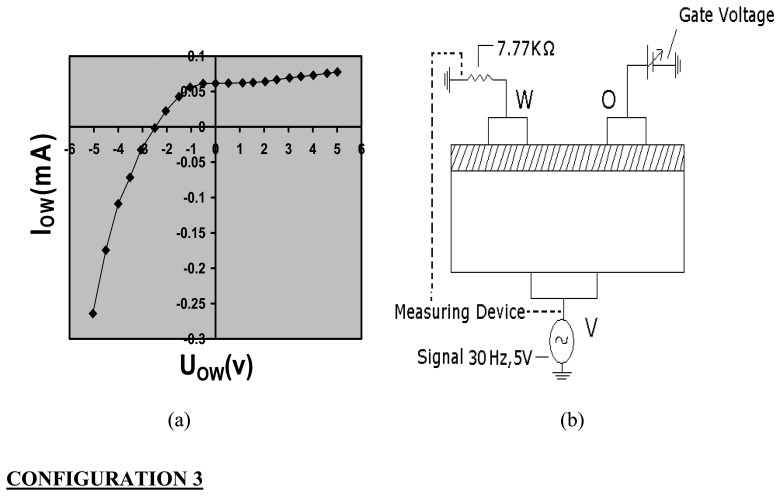
(a) Current/voltage characteristics of PEO:NH_4_ClO_4_+CdS based TEMPOS structures in configuration 3; (b) the principle measuring setup for current/voltage determination in configuration 3.

Without exposure to ammonia and/or water, the TEMPOS structures behave as diodes (Figures 2,3 and 4) in the three terminal (source-drain-gate) configuration when measured from the top to the bottom contact.

This diode-like behaviour stems from the band transition of the track material (mixed conductor) and the semiconducting substrate. When used as a two terminal device (gate voltage being zero or gate terminal floating, Figure 3c), the I–V characteristics show hystereses due to the polarization of the polymer electrolyte under an applied electric field.

**Figure 5.**
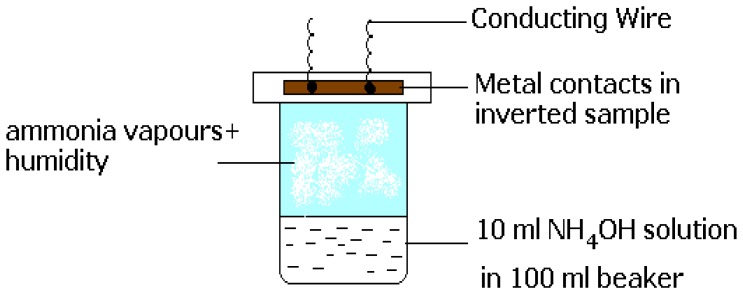
Schematic diagram of the beaker filled with a liquid (water or ammonium hydroxide) and the sample exposed to it.

The measurements were carried out over the frequency range 1Hz – 100 KHz with voltages varying from 1 to 5 V. Each measurement corresponds to the average value of 64 readings. The whole system was kept in a probing vessel (as shown in Figure 5) at room temperature (28° C) and care was taken to record readings under identical environmental conditions.

Measurements were first recorded with pure water in the probing vessel and then with various concentrations of ammonia solution leaving the sample to equilibrate for 24 hours in the new atmosphere. With each change in voltage, the system was again left to stabilize for at least 30 min. In total, four samples were studied using always the same polymer electrolyte (PEO/NH_4_ClO_4_). Whereas one sample was used as prepared, in the other three cases ZnS, PbS and CdS nanoparticles were dispersed in the polymer electrolyte.

**Figure 6.**
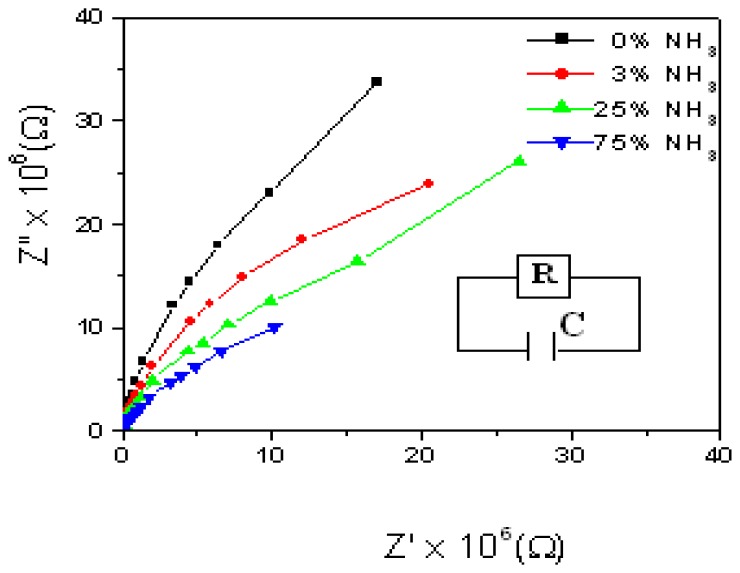
Cole-Cole plots of CdS dispersed PE based TEMPOS structures at different NH_3_ concentrations (inset: equivalent circuit).

The Cole–Cole plots clearly show (Figure 6) that with increasing concentration of ammonia solution, the radius of curvature of the arcs decreases, thereby indicating a decrease in resistance. The impedance change takes place over a wide range of ammonia concentrations. Mass transport leads to accumulation of ions at the electrodes that tend to increase the impedance. Hence, a proper choice of operating frequency, high enough to avoid ion accumulation, is essential for the optimum sensor performance.

**Figure 7.**
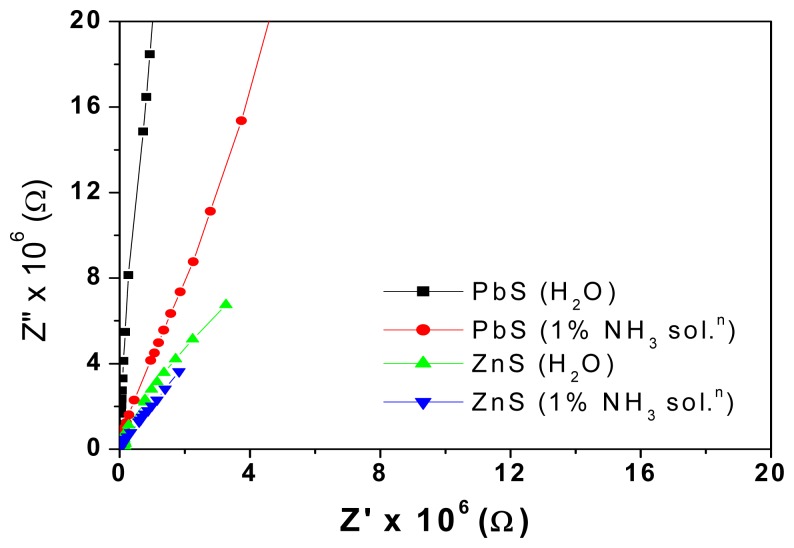
Cole-Cole plots of PbS dispersed PE and ZnS dispersed PE based TEMPOS structures with water and 1% ammonia solution.

Ammonium hydroxide dissociates to give both ammonia gas and water vapour. In order to bring out the contribution of both vapours, impedance measurements were first taken for samples exposed to the same volume of water and then for various concentrations of ammonium hydroxide solution. Figure 7 clearly shows the arcs of smaller radii when exposed to ammonium hydroxide solution as a higher number of protons is available for conduction.

**Figure 8.**
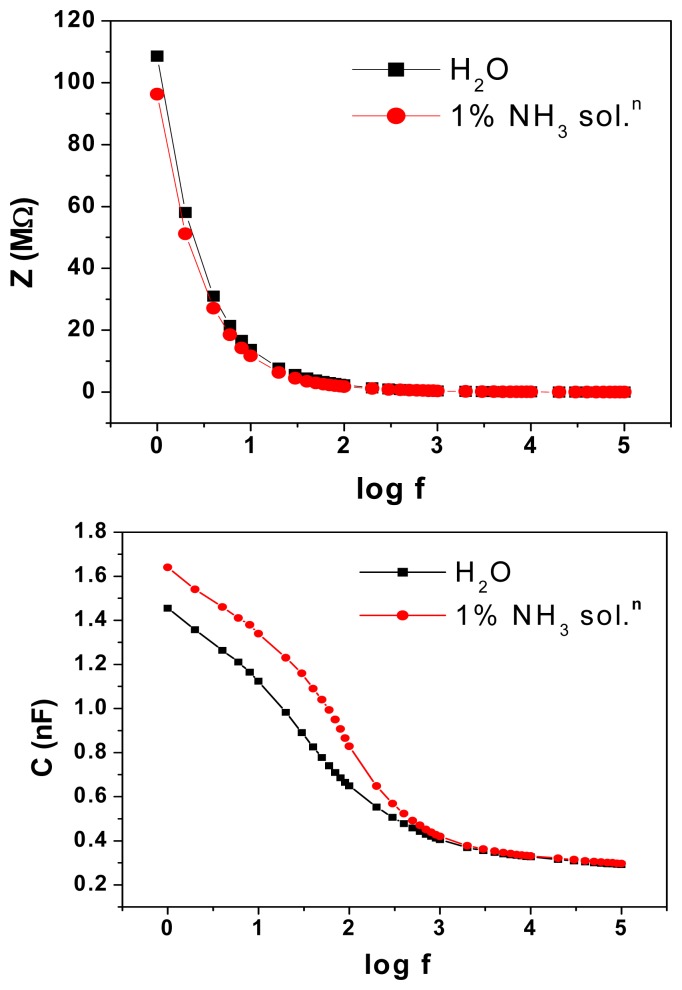
(a) Variation of impedance and (b) capacitance with frequency of polymer electrolyte based TEMPOS structures on exposure to water and also to 1% NH_3_ solutions.

Figures 8a and 8b show the manner in which impedance and capacitance vary with frequency in polymer electrolyte based TEMPOS structures when exposed to water and 1% ammonium hydroxide solution, respectively.

**Figure 9.**
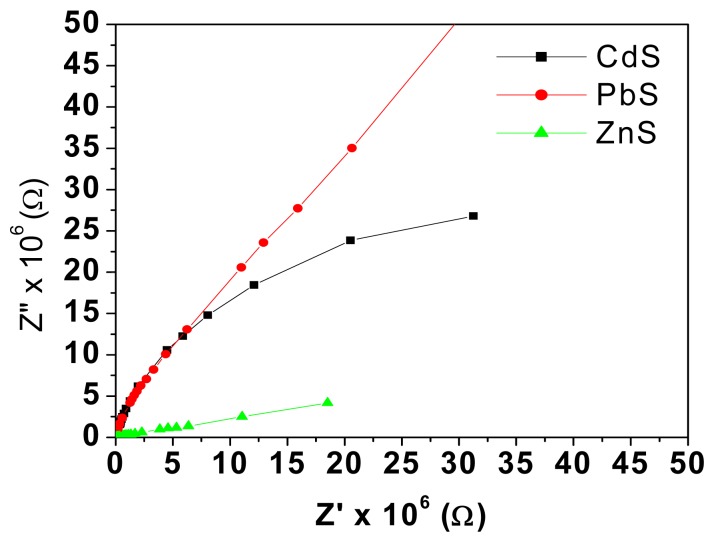
Cole-Cole plots of mixed conductor filled TEMPOS structures for 3% NH_3_ solution.

Impedance values are smaller and the capacitance is larger on exposure to ammonia vapour than to water. The large dielectric constant of water contributes to this. At high frequencies, the water dipoles are not able to cope up with the fast change in direction of the applied signal and hence attain a constant value. Figure 9 shows the behaviour of the mixed conductor filled TEMPOS structures on exposure to 3% ammonia solution.

**Figure 10.**
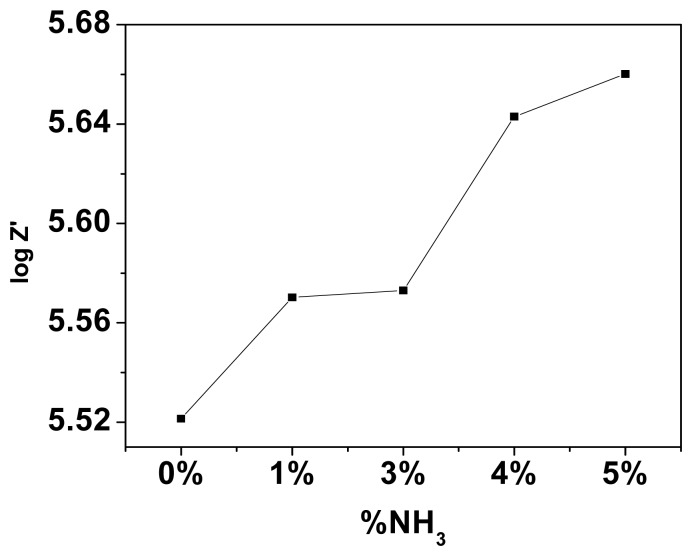

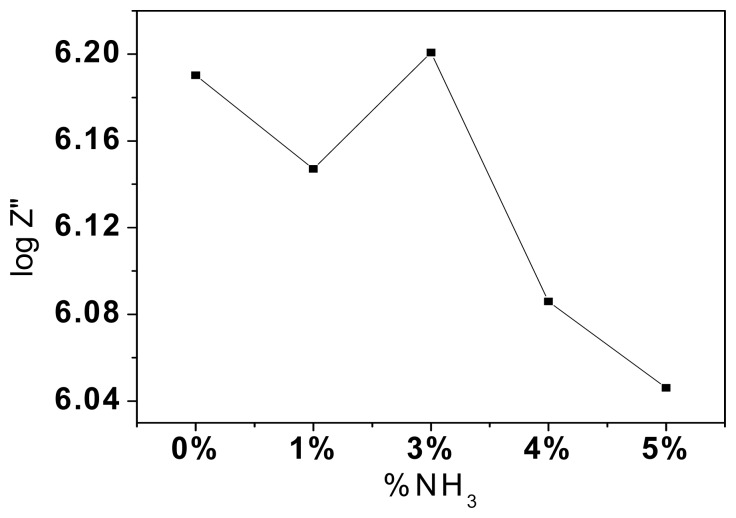
(a) Variation of the real and (b) imaginary parts of impedance with NH_3_ % at 100 Hz of PEO: NH_4_ClO_4_+ CdS based TEMPOS.

Figures 10a and 10b show both the variation of real (resistance) and imaginary (capacitance) part of impedance as a function of ammonia concentration of a mixed conductor based TEMPOS.

**Figure 11.**
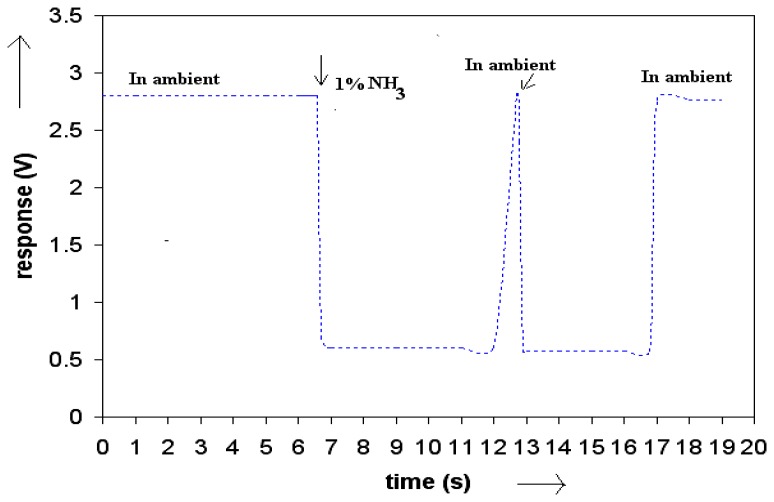
Response-time curve for mixed conductor based TEMPOS structures.

Figure 11 gives the response time of CdS dispersed PE inserted in TEMPOS structure on exposure to 1 % NH_3_ solution. The gate voltage has been taken as 2V for measurement as the TEMPOS structure behaves as a diode around this gate voltage. It is observed that the response time of the sensor is ∼0.2 seconds.

The gas sensing behaviour of TEMPOS structures is a cumulative response of the silicon dioxide [38], the polymer electrolyte and the mixed conductors embedded in the etched tracks. The conduction along the tracks of the TEMPOS sensors studied here exceeds the inter-track conduction. Therefore, the voltage applied to the third contact has little influence on the TEMPOS sensing behaviour.

## 3. Experimental Section

### 3.1. Sample preparation

Figure 1 shows the schematic diagram of a TEMPOS structure. It is similar to the classical MOSFET structure except that there are tracks in the dielectric layer. The number and dimensions of these tracks depend on the ion energy, ion fluence, ion charge and the target material. In the present study, the mixed conductor has been filled in the tracks. The TEMPOS configuration in the present study was as follows: p-Si/SiO_2_/PEO:NH_4_ClO_4_ +CdS (96:4+5 wt.% of Cd-salt)

Other corresponding salts were dispersed to get PbS and ZnS based mixed conductors. The pores, such as etched ion tracks, can be filled with material by many techniques such as drying of an embedded solution, pressure injection, galvanic deposition, chemical deposition, evaporation, etc. [39]. One of the easiest approaches towards technological applications is to fill the pores within the dielectric with some appropriate material that can serve as a sensor. In order to increase the sensitivity by a large surface of the sensitive material, it is usually desirable in this case, to deposit the material as a thin film into the pores' inner walls, thus forming nanotubules. This geometry also enables rapid access of the material to be probed to the sensor material so that the resulting device has fast response times.

In the present study, Si wafers with thickness of a few hundred micrometer, were obtained commercially. Subsequently, the samples were oxidized in dry atmosphere at 1200°C to obtain SiO_2_ layers of ∼100 nm thickness. Thereafter, the sample was exposed to 350 MeV Au^26+^ions at fluence 10^9^ions/cm^2^. The applied fluence was below the onset of ion track overlapping. The tracks in the oxide layer were etched with 0.55% HF solution for ∼ 14 minutes and then rinsed in de-ionized water. The etchant preferentially attacks the ion–irradiated zones and leaves the un-irradiated oxide and the silicon unaffected. The etching time determines the size and shape of the pores.

A methanolic solution of mixed conductor was inserted into the tracks. The mixed conductors used in the present study are 5 wt.% Cd (Pb or Zn) salt dispersed in PEO:NH_4_ClO_4_ (96:4). A syringe was used to insert the solution in several steps drop by drop onto the sample and letting it dry. This procedure resulted in the dispersion of the semi-conducting nano-crystals on the ion track walls. Finally, three contacts were made with conducting silver paste and the sample was left to dry (Figure 1)

### 3.2. The measuring setup

Usually, TEMPOS test structures are constructed such that they have one back contact ***v*** and two equidistant surface contacts ***w*** and ***o*** which are spaced from each other by few millimeters, here, 7 mm. This three contact design resembles the classical bipolar or field effect transistor arrangement: (emitter-base-collector) or (source-drain-gate), respectively. In contrast, in TEMPOS structures, one has the freedom to define any one of the 3 contacts, o, v or w as source, drain or gate (emitter, base or collector) respectively, as all contacts are coupled both conductively and capacitively to each other. AC voltage of ∼ 30 Hz and 5V was applied for the current – voltage study. The current response through the device with changing gate voltage in all the three configurations (Figures 2,3-4) was recorded by means of a Keithley multimeter. The calibration of the sensor response to ammonia is performed using the setup given in Figure 5. To check for the influence of H_2_O vapour, its response has been studied.

At first, the samples were exposed to water of constant volume and Cole-Cole plots were taken. Thereafter, ammonia solution (of same volume) was taken and the sample had direct exposure to the ammonia vapours. The whole set up was kept inside an air tight chamber and Cole-Cole plots were made at 1 to 5V for various concentrations of ammonia solutions (0%, 1%, 3%, 25% and 75%) and frequencies ranging from 1 Hz to 100 KHz.

## 4. Conclusions

With increasing concentration of ammonia solution, the radius of curvature of the arcs (Cole-Cole plots) decreases, thereby indicating a decrease in resistance. The impedance change takes place over a wide range of ammonia concentrations.

Ammonium hydroxide dissociates to give both ammonia gas and water vapour. The arcs of smaller radii when exposed to ammonium hydroxide solution are due to the higher number of protons available for conduction. Impedance values are smaller and the capacitance is larger on exposure to ammonia vapour than to water. The large dielectric constant of water contributes to this. At high frequencies, the water dipoles are not able to cope up with the fast change in direction of the applied signal and hence attain a constant value. The gas sensing behaviour of TEMPOS structures is a cumulative response of the silicon dioxide, the polymer electrolyte and the mixed conductors embedded in the etched tracks. Sensitivity of sensors depends on frequency, material in tracks and voltage of applied signal. The set up is inexpensive and working temperature is room temperature. Table 2 gives a summary of the parameters of different types of ammonia sensors and sensor systems.

## Figures and Tables

**Table 1.** Comparison of ammonia sensors.

**NH_3_phase to be detected**	**Detector type**	**Working medium**	**References**
Gaseous	Insulating polymer	Bisphenol-A-polycarbonate	[19]
	Conducting polymer	Polyamide	[5, 7]
		Nafion	[8]
		Polypyrrole	as-received [9] SHI modified [19]
	Metal oxide	SnO_2_	[10, 11 ]
		CdSnO_3_	[12]
		TiO_2_	[13]
		In_2_O_3_	[14]
	Catalytic (based on change in charge carrier conc. in the metal)	Pd	[6]
	TEMPOS	PEO:NH_4_ClO_4_-filled etched tracks in SiO_2_ or SiON on Si	This work
Liquid	Optical (Spectrophotometric NH_3_ detection based on pH change)	Nessler reagent (dipotassium tetraiodomercurate in NaOH) Berthelot reagent (phenol + hypochlorite)	[6]

**Table 2.** Parameters of different types of ammonia sensors and sensor systems.

**Sensor type**	**(Ir)reversibility**	**Detection threshold**	**Response time[s]**	**Remarks**	**Working temperature (°C) and structure**

Conducting polymer (PPy)	Irreversible	10^-6^	∼ 250	Sensitivity decreases with time	Up to 150°C
Metal oxides (WO_3_)	Reversible	10^-6^	∼300	Low selectivity drift	400°C, rugged, inexpensive
Catalytic (Palladium)	Reversible	10^-6^	∼ 60	Limited accuracy, selectivity depends on temperature	Up to 600°C
Optical: (Nessler)	Irreversible	10^-9^	60	All are sensitive and selective and have expensive setup	37°C
(Coulorometric)		10^-12^	300		
(Absorption spectroscopy)		10^-9^	300		
SHI-modified IPCP	Reversible	10^-2^	∼500	Sensitivity ion-fluence dependent	Room temp. (25-28°C), inexpensive set up
This work: TEMPOS sensor	Reversible	∼10^-5^	∼0.2	Sensitivity depends on frequency, material in tracks and voltage of applied signal	Room temperature (28°C), inexpensive set up
